# Discrimination of Myrtle Ecotypes from Different Geographic Areas According to Their Morphological Characteristics and Anthocyanins Composition

**DOI:** 10.3390/plants8090328

**Published:** 2019-09-05

**Authors:** Ana V. González-de-Peredo, Mercedes Vázquez-Espinosa, Estrella Espada-Bellido, Marta Ferreiro-González, Antonio Amores-Arrocha, Miguel Palma, Gerardo F. Barbero, Ana Jiménez-Cantizano

**Affiliations:** 1 Department of Analytical Chemistry, Faculty of Sciences, University of Cadiz, Agrifood Campus of International Excellence (ceiA3), IVAGRO, 11510 Puerto Real, Cadiz, Spain (A.V.G.-P.) (M.V.-E.) (E.E.-B.) (M.P.) (G.F.B.); 2 Department of Chemical Engineering and Food Technology, Faculty of Sciences, University of Cadiz, Agrifood Campus of International Excellence (ceiA3), IVAGRO, 11510 Puerto Real, Cadiz, Spain (A.A.-A.) (A.J.-C.)

**Keywords:** anthocyanins, chemometric methods, hierarchical cluster analysis, morphological parameters, *Myrtus communis* L., phenolic compounds, principal component analysis

## Abstract

*Myrtus communis* L. is an evergreen shrub that produces berries with a high content in antioxidant compounds. Since these compounds have demonstrated a positive effect on human health, the interest on berries and their usages has increased. However, environmental conditions may affect the productivity of these species and consequently the quality of wild myrtle. Ecotypes from diverse geographical origins may result in significant variations in terms of bioactive compounds content as well as in chemical traits. For this reason, in this work ecotypes from two different localizations have been studied to determine if their differences in morphological and anthocyanins traits can be attributed to their origin and the environmental characteristics of these locations. For this, chemometric analyses such as Hierarchical Cluster Analysis and Principal Component Analysis, were employed. The results showed differences between the ecotypes depending on their location. In particular, myrtle berries from maritime zones present greater fruit size and amount of bioactive compounds, which means an improvement in the quality of the final product based on this raw material. It can be concluded that both morphological and anthocyanins traits are related to the location of the ecotype and allow selecting the best ecotype for the required applications.

## 1. Introduction

*Myrtus communis* L., common myrtle, is a plant that belongs to the Myrtaceae family. It comprises approximately 145 genera and over 5500 species [1], myrtle being one of the most important aromatic and medicinal species from its family. Myrtle is a common species in typical Mediterranean flora and grows naturally in Iran, Spain, France, Greece, Turkey, Algeria, Morocco, Croatia, Italy and Montenegro [2,3]. It is a 0.5 to 6 m tall evergreen shrub characterized by the following vegetative features: opposite leaves, rough bark, white flowers (five to nine petals) and berries of variable color (white, purple, blue, or black) [4] and form (spherical, elliptic, obovate, pyriform, or turbinate) [5]. 

Ancient Mediterranean populations largely used myrtle due to its ornamental and aromatic value [6]. This species is very aromatic because of the high essential oil content in its leaves, flowers and fruit glands [7]. Nowadays, the species has gained increasing interest for the high content in bioactive compounds in its berries, mainly phenolic compounds such as anthocyanins [8,9]. Numerous studies support a positive association between the concentration of phenolic compounds in the berries and preventive properties against different diseases, such as cardiovascular or neurodegenerative diseases [10,11,12]. Based on these properties, cosmetics, medicine and food industries have found new application fields for myrtle berries [13]. For example, Sardinian myrtle liqueur, the typical spirit obtained from myrtle leaves and berries by alcoholic maceration, has recently experienced a growing demand in the market [14]. In the same way, new studies that intend to develop fast and efficient methods to extract and analyze phenolic compounds and anthocyanins contents in myrtle are being published [15,16].

In regard to the healthy properties of this species, the selection of the appropriate myrtle ecotype is also very important [17]. For instance, myrtle ecotypes with higher amounts of phenolic compounds allow elaborating myrtle liqueurs with better color parameters and superior organoleptic and healthier properties [18]. Environmental factors (temperature, rainfall, climate or soil) have a relevant effect on the productivity of the plants [19]. In fact, plants of the same species may show differences in their bioactive compounds content and morphological traits because of their differences in growing environmental conditions [20,21]. Thus, in order to select the appropriate ecotype, it is necessary to have a thorough knowledge of the morphological characters and the chemical composition that result from each different ecotype. 

In order to determine its chemical composition, adequate extraction methods that extract the bioactive compounds of interest from natural matrices like myrtle’s must be developed. Microwave-Assisted Extraction (MAE) is one of the advanced extraction methods more widely used [22]. It is considered to be a promising green extraction method that reduces both extraction time and solvent consumption [23,24]. MAE was optimized and recently published by the authors for the extraction of Total Phenolic Compounds (TPC) and Total Anthocyanins (TA) from the same myrtle berries [25]. With respect to how much is known about its morphological characteristics, particularly in Spain, the variability of morphological traits between wild myrtle populations has not been deeply studied yet. According to the bibliography, the morphological traits usually analyzed in myrtle are the following: fruit length, fruit width, fruit weight, peduncle length, fruit shapes, calyx shapes, calyx diameter, seed number, seed length, seed width, seed weight, total seed weight, leaves traits, leaf length, and leaf width [26].

Although the interpretation of the results is still a challenge [27], in the last decade new alternatives or complementary approaches have been studied. Chemometrics is the chemical discipline that uses mathematical, statistical, and other methods to provide maximum relevant chemical information by analyzing chemical data [28]. The chemometric tools have been widely employed with a large variety of matrices such as wine [29], cabbage [30], mulberry [31], and honey [32]. Regarding myrtle berries, according to the bibliography the most adequate chemometric tools to determine whether the myrtle traits evaluated are related to the location of the ecotype, are hierarchical cluster analyses (HCA) and principal component analysis (PCA) [20].

Therefore, the aim of this work was to evaluate the effect of the geographical origin and environmental conditions in the final morphological and anthocyanins traits of two different wild myrtle population from two areas (coastal and inland) with different climatic characteristics in the province of Cadiz (Andalucía, Spain) by using chemometric tools (HCA and PCA).

## 2. Results and Discussion

### 2.1. Morphological Description

Results of morphological traits of fruits, seeds and leaves are represented in Table 1. The average variability of the morphological traits was expressed by the coefficient of variation (CV). The fruit length of the selected ecotypes varied from 8.80 mm to 16.24 mm, and the fruit width varied from 6.18 mm to 11.29 mm. The minimum coefficient of variation and therefore the least variability was found for fruit length (CV = 8.69%). However, fruit width showed greater variability among the ecotypes, with wider fruits in the ecotypes collected in Puerto Real. Furthermore, these ecotypes also had the highest number of seeds. The maximum coefficient of variation and therefore the highest variability was found for seed number (CV = 40.86%), which varied from 1 to 10. On the other hand, these ecotypes also showed minor values of width seed and weight seed. This ranged from 2.50 to 4.47 mm and 0.0106 to 0.0240 g, respectively. Differences among populations were less clear for leaf traits. Leave length ranged from 28.25 to 46.21 mm and leave width between 10.55 to 18.45 mm. With respect to categorical variables, the form of the berries varied depending on the ecotype, but all showed blue fruit color. 

### 2.2. Correlations between the Morphological Traits

The correlation coefficient is a relevant statistical method to quantify associations between variables. Specifically, the correlation between morphological variables was established using Pearson’s chi-square test. This analysis provides information on the trends and relations between the morphological variables in the genotypes studied. Later on, this data was complemented by a HCA and PCA analysis for a better understanding of the trends. The correlations showed significant values for some of the morphological characters (Table 2). In the literature, Wahid et al. [26] have previously reported the correlations between morphological variables obtaining similar correlations results. In the present study, quantitative traits revealed a high and significant correlation between fruit width and fruit weight (r = 0.874). In this sense, Wahid et al. [26] revealed a similar significant correlation (r = 0.775); however, these authors also showed that both traits (fruit width and fruit weight) showed correlations with fruit length. These correlations are not revealed in the present study.

Regarding the seeds, both fruit width and weight were correlated with seeds number (r = 0.845, r = 0.738; respectively) and total seeds weight (r = 0.695, r = 0.814; respectively). Moreover, a positive correlation between seeds length and seeds width (r = 0.689) was found. Wahid et al. [26] revealed the same correlations but more moderate, with correlation values around r = 0.3. Moreover, both, seed length and width were correlated with seeds weight (r = 0.849, r = 0.730; respectively). This correlation was not revealed by the aforementioned authors [26].

Finally, with respect to leaves, a significant and highly positive correlation was observed between leaf length and leaf width (r = 0.821). Similarly, a highly positive correlation was observed between leaf length and leaf width (r = 0.808) by Wahid et al. [26].

### 2.3. Correlation between the Morphological and the Chemical Traits

As in the previous case, the correlations between morphological variables and chemical variables was established by means of Pearson’s chi-square test. Specifically, the variables employed in this study were all the morphological variables (FL, FWD, FWG, PL, CD, SN, SL, SWD, SWG, TSWG, LL, and LW) and as chemical traits, the TPC and the TA. The TPC and TA obtained by MAE are represented in a previous study [25]. The correlations showed significant values for some of the variables (Table 3). Anthocyanins are a subclass of phenolic compounds, therefore, both chemical parameters are correlated to each other and both have, in most cases, a significant correlation with the same morphological traits.

The quantitative traits of myrtle’s berries revealed a positive correlation between the TA and berries’ width (r = 0.679), seeds number (r = 0.598), and total seeds weight (r = 0.562). Moreover, the TA was negatively correlated with fruit length (r = −0.541). These results indicate that wide and not very long myrtle berries with a large number of seeds have a higher concentration of TA. With respect to TPC, this variable was correlated with the same morphological traits with the exception of fruit length and fruit width. The results obtained confirm that the study of morphological traits helps to select the best ecotype for each different application (industries, laboratories, etc.). By only determining the morphological traits of each myrtle ecotype, it is possible to know which myrtle ecotypes present a greater amount of TA and TPC without the need of a previous chemical analysis.

### 2.4. Correlation between Morphological Traits, Chemical Traits and Geographic Location

For this study, the morphological and chemical features data were grouped separately, and the average values were used.

#### 2.4.1. Exploratory Chemometric Study of Morphological Data

**Figure 1:**
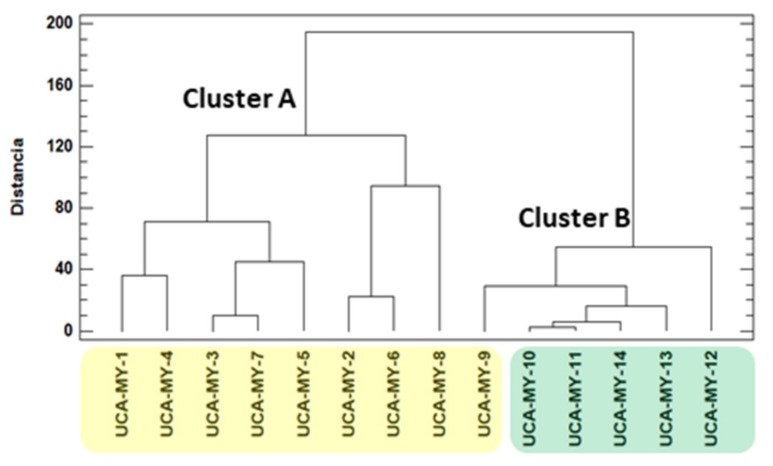
Dendrogram obtained by hierarchical cluster analysis (HCA) of the morphological parameters studied (D_14 × 12_).

Based on the morphological data, it can be observed that there are noticeable differences between the average values corresponding to the different myrtle ecotypes. For this reason, to objectively study if these apparent differences are related to the location of the ecotypes as well as to the environmental characteristics of these locations, a comparative chemometric study was carried out using all the average morphological values. Firstly, HCA was applied to the whole data matrix (D_14 × 12_) [33]. HCA is a non-supervised chemometric tool that allows uncovering unknown trends in the data regarding the grouping of the myrtle ecotypes. The results obtained from the analysis of hierarchical clustering with Euclidean distance using the Ward method are shown in the dendrogram in Figure 1.

The results show a grouping trend that is related to ecotype location. There are two main groups: group one (Cluster A) includes all the myrtle ecotypes collected from Puerto Real area, and group two (Cluster B), that includes only myrtle ecotypes collected from San José del Valle area.

**Figure 2:**
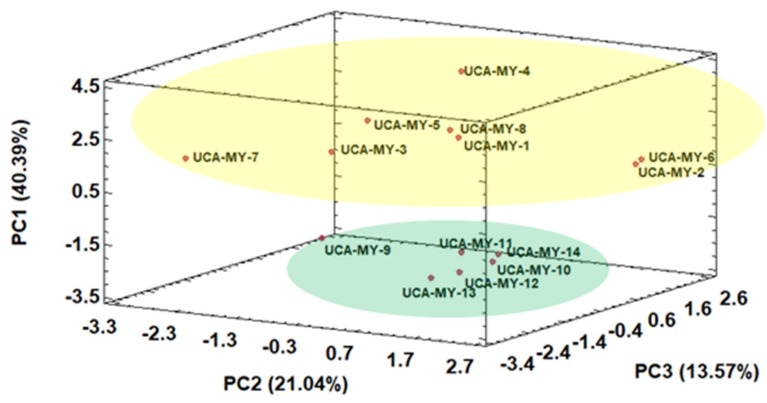
Three-dimensional score plot for the first three principal components based on morphological characters (average values, *n* = 25) (D_14 × 12_).

Thus, a perfect separation of the ecotypes in two groups by means of HCA was obtained. Then, another non-supervised technique namely PCA was applied to the same data matrix D_14 × 12_. Thanks to PCA the spatial classification of the ecotypes and the variables that take part in the classification can be observed. Kaiser criterion was applied to decide the number of principal components (PCs) to be extracted. According to Kaiser criterion, during a standardized PCA, only those components whose eigenvalues are greater than 1 must be maintained [34]. Specifically, a total of 4 PCs were obtained and just the first three components were enough to produce a successful classification as shown in Figure 2.

The results obtained from the PCAs showed the potential to discriminate between the ecotypes mainly due to PC1. On the one hand, the myrtle ecotype samples collected from Puerto Real area showed higher PC1 values, all in the positive area of PC1. On the other hand, the myrtle ecotype samples collected from San José del Valle area showed lower values for PC1, all in the negative area of PC1. These results suggest that there is a tendency of the ecotypes to be classified on the space based on their origin. From Figure 2 it was also observed that the ecotype samples from Puerto Real area showed a wider variability and formed a more heterogenous group than the ecotype samples from San José del Valle. 

Table 4 shows the normalized loadings parameters, which reflect the relevance of the variables with respect to each PC. PC1 represented 40.39% of the total variance and was positively related to fruit width and seeds number, while it was negatively related to seed width and seed weight. This means that the ecotype samples from Puerto Real (with high PC1 values) generally have wider fruits with a greater number of seeds, which agrees with the results obtained from the Pearson correlation analysis, where these two morphological variables were correlated with each other. Furthermore, the seeds from Puerto Real ecotype samples are narrower and lighter than the seeds from the ecotype samples from San José del Valle (with low PC1 values), which also agrees with the high correlation between seeds width and weight. Therefore, it can be concluded that the morphological information obtained is related to ecotype location, since there is a tendency to form groups according to their geographical origin. It can be seen that the higher relative humidity in Puerto Real area and its soils with a greater water content leads to myrtle ecotypes with a greater fruit size. This is because the climate in Puerto Real provides more fertile conditions for the growth and ripening of myrtle ecotypes. 

#### 2.4.2. Exploratory Chemometric Study of Chemical Data

Based on the chemical results, an additional chemometric analysis was carried out to determine if, in the same way as with morphological results, the chemical information obtained from bioactive compounds (phenolics and anthocyanins combination) was also related to the geographical origin of the ecotypes. To this aim, the chemical results obtained by means of MAE in a previous study [25] were analyzed by HCA and PCA using the whole data matrix (D_14 × 13_). The variables that were taken into consideration to form the groups were TPC, TA, and 11 individual anthocyanins. 

**Figure 3:**
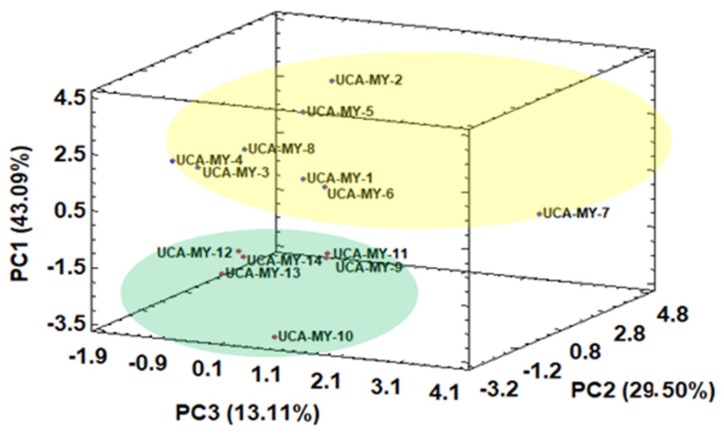
Three-dimensional score plot for the first three principal components based on chemical characters (average values, *n* = 2) (D_14 × 13_).

Firstly, similarly to the procedure employed for the morphological results, HCA was applied to the data (D_14 × 13_). The results obtained from the HCA are collected in an article previously published by the authors where two main groups can be observed [25]: Group one (Cluster A) which includes all the myrtle ecotype samples from Puerto Real, and Group two (Cluster B), that only includes myrtle ecotype samples from San José del Valle. Once it was clear that, by means of HCA, a total separation in two ecotype groups was obtained, PCA was applied to the same data matrix D_14 × 13_. The score plot of all the myrtle ecotypes according to the first three PCs obtained by means of PCA is shown in Figure 3.

A general tendency to separate myrtle ecotypes from different locations can be also observed when MAE was employed. The results obtained by PCA show the feasibility to discriminate between ecotypes just by PC1. Specifically, the myrtle ecotype samples collected from Puerto Real showed positive PC1 values, while the ecotype samples collected from San José del Valle showed low negative PC1 values. In both groups, there is one ecotype (UCA-My-7 from Puerto Real and UCA-My-10 from San José del Valle) more heterogeneous than the rest of the ecotypes in the same group. Table 5 shows the normalized loadings parameters, which reflects the relevance of the variables with respect to each PC. 

PC1, which represented 43.09% of the total variance, was positively related to TPC and TA. With regard to individual anthocyanins, PC1 was mainly related to delphinidin 3,5-*O*-diglucoside, and delphinidin 3-*O*-glucoside, the latter being the main anthocyanin found in myrtle. This means that the ecotypes from San José del Valle (with low PC1 values) generally have a lower amount of TPC and TA than the samples collected from Puerto Real (with high PC1 values). For example, berries from Puerto Real showed a high level of delphinidin 3,5-*O*-diglucoside, and delphinidin 3-*O*-glucoside. This is of great interest due to the fact that anthocyanin contents as pigments have substantial effects on the preservation of the quality of many fresh and processed fruits, vegetables, groceries as well as plants’ products [35]. These results are consistent with literature information, which highlights that myrtle is a shrub that prefers fertile and humid soils like the ones found in Puerto Real, where it grows better and produces greater amounts of biological compounds [36]. 

#### 2.4.3. Influence of Ecotype Location on Morphological and Chemical Characteristics

Although *Myrtus communis* L. has proven its capacity to adapt to different environments [37], the chemometric analysis showed how some of the morphological and chemical characteristics of its different ecotypes vary according to the geographic area where they grow. These differences may be due to variations in the environmental characteristics, since the physical (i.e., rainfall, temperature) and soil factors (i.e., soil quality) influence the available resources and, therefore, may affect myrtle berries’ phenolic composition and morphological characteristics [19,38,39]. In this sense, the myrtle ecotypes from Puerto Real area showed, among other characteristics, greater fruit size and a higher content in phenolic and anthocyanin compounds than the ecotypes from San José del Valle. Therefore, individual plants of the same species grown under different environmental conditions could present significant variations in secondary metabolites content and morphological characteristics [40]. This can be also very useful, i.e., for producers since they can select the myrtle ecotype based on their final goal. For example, myrtle liqueurs elaborated with maritime myrtle berries showed better color parameters and superior organoleptic properties. Furthermore, these liquors will present better medicinal properties [41].

## 3. Materials and Methods 

### 3.1. Plant Material and Study Area

The plant materials employed in this work were myrtle berries collected by the authors between 2016 and 2017 at their optimum ripeness stage (November). The berries, collected from different Cadiz ecotypes, were stored in a freezer prior to analysis (−20 °C). The study has been focused on two areas of the province of Cadiz (Andalusia, Spain): Puerto Real and San José del Valle. Although both regions are in the province of Cadiz, they have different climatic characteristics mainly due to their different distance to the sea shore. While Puerto Real is a coastal region, the region of San José del Valle is located 50 km inland. Proximity to the coast results in very humid environments. Therefore, in Puerto Real there is a higher relative humidity in the environment throughout the year, even in the summer. So, this region is characterized by abundant water content in its soils. On the other hand, San José del Valle is an inland zone with a wider temperature variation and lower water content available for the plants, especially during the summer months. The weather conditions in each area were obtained from meteorological stations and the data registered are shown in Table 6.

**Figure 4:**
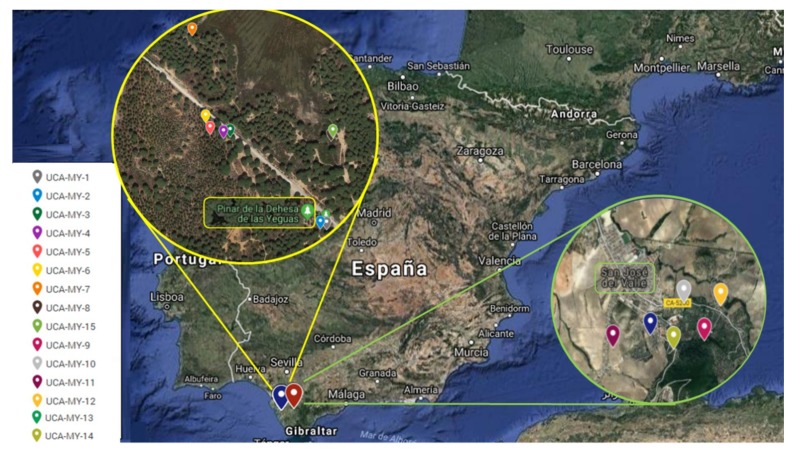
The geographic location of the study areas in Cadiz (Andalusia).

Specifically, samples from eight different ecotypes were collected from Puerto Real shrubs and from six different ecotypes from San José del Valle shrubs. The shrubs were randomly chosen without any phenotypical selection and at least 50 m apart from each other to avoid sampling from related individuals [26]. From each shrub, 25 berries and 25 leaves were randomly collected to determine their traits. The geographic locations of the 14 ecotypes are shown in Table S1 and Figure 4. 

### 3.2. Morphological Analysis

Different morphological characteristics were studied using the descriptors list proposed by Mulas and Cani (1999) as reference [37]. Table 7 shows the information of the different morphological traits (with its codes) and the methods of measurement.

Altogether, 14 morphological traits including 12 quantitative characters (fruit length, fruit width, fruit weight, peduncle length, calyx diameter, seed number, seed length, seed width, seed weight, total seed weight, leaf length, and leaf width), and 2 qualitative characters (fruit shapes, and calyx shapes) were separately noted down for each ecotype sample collected. Their fruit colors were disregarded as morphological traits in the subsequent chemometric analysis since all the ecotypes collected showed blue berries. The data were evaluated on 25 berries and 25 leaves selected randomly from each ecotype.

### 3.3. Chemical Analysis

In total, 13 chemical traits including TPC, TA, and 11 individual anthocyanins were recorded individually for each ecotype sample collected. It was decided to study only anthocyanins individually because they are the majority phenolic compounds found in myrtle [42]. 

The extraction of TPC and TA was carried out employing the MAE methods recently published by the authors [25]. Different conditions were employed for the extraction of anthocyanins and for the extraction of phenolic compounds from myrtle berries according to the results obtained through a Box–Behnken design (BBD) with a response surface methodology (RSM). 

With respect to the anthocyanins, they were extracted from 0.5 g of the sample by MAE (800 W) with 20 mL of water–methanol (50.4% methanol) acidified (pH 3.33) at 50 °C for 2 min. Once the compounds were extracted, they were analyzed. The anthocyanins were first identified using ultra-high-performance liquid chromatography (UHPLC) coupled to quadrupole-time-of-flight mass spectrometry (Q-ToF-MS). Then, they were quantified employing high-performance liquid chromatography (HPLC). Specifically, a rapid HPLC method with a C18 reverse-phase fused-core column, previously described in the bibliography [43], was employed. The following eleven anthocyanins were identified in the samples: delphinidin 3,5-*O*-diglucoside, delphinidin 3-*O*-glucoside, cyanidin 3-*O*-galactoside, cyanidin 3-*O*-glucoside, cyanidin 3-*O*-arabinoside, petunidin 3-*O*-glucoside, delphinidin 3-*O*-arabinoside, peonidin 3-*O*-glucoside, malvidin 3-*O*-glucoside, petunidin 3-*O*-arabinoside, and malvidin 3-*O*-arabinoside. 

With respect to the phenolic compounds, they were extracted from 0.5 g of the sample by MAE (800 W) with 20 mL of acidified water–methanol (58.20% methanol; pH 2) at 100 °C for 15 min. Finally, for the quantification of TPC the Folin–Ciocalteau spectrophotometric method was employed [44,45]. 

### 3.4. Data Analysis

All the samples (*n* = 14) were analyzed in duplicate for chemical analysis and 25 times for morphological analysis. Means and CV were evaluated for all the measured morphological traits. The CV were determined as indicators of variability. The correlations between variables were calculated using Pearson’s chi-square test for 2 × 2 contingency tables. Furthermore, multivariate analysis of the data, which include non-supervised chemometric tools such as HCA and PCA, were carried out. The objective of HCA is to classify a series of individuals in groups (clusters) according to their similarity and dissimilarity. On the other hand, PCA is a multivariate technique in which a number of related variables are transformed into a set of uncorrelated variables called principal components (PCs). So, PCA allows reducing the dimensionality of large data sets, by transforming a large set of variables into a smaller one [34]. The data for the morphological and chemical characters were grouped separately and the data of each group were arranged in matrixes named D_m × n_ where *m* is the number of myrtle ecotypes and *n* is the number of variables. For all the different multivariate analysis carried out, the mean of each character was normalized by assigning one unit to the maximum value. Statgraphic centurion XVII software (Statgraphic Technologies, Inc., The Plains, VA, USA) was used for all chemometric analyses. 

## 4. Conclusions

To the best of our knowledge, this is the first study in which the variability of morphological traits between wild myrtle populations has been studied in Spain. Different ecotypes from two locations in Cadiz, Spain (coastal and inland) were collected to evaluate morphological and chemical traits differences between myrtle ecotypes grown under different environmental conditions. Based on the results obtained after applying the chemometric techniques (HCA and PCA), it can be concluded that both the morphological and chemical information is related to the location of the ecotypes due to the tendency to be grouped according to their geographical origin. Such variations in berries’ chemical composition and morphological characteristics between the different ecotypes could be a useful hint for the selection of the *Myrtus communis* L. ecotypes that present the most desired traits for each particular purpose. In general, according to the chemical analysis data obtained in this study, it could be said that it is more convenient to select myrtle berries from maritime zones, since they present a greater amount of bioactive compounds such as anthocyanins. This information could lead to an improved quality of the products elaborated from this raw material. 

## Figures and Tables

**Table 1:** Descriptive analysis of morphological traits of 14 natural ecotypes of *Myrtus communis* L. The table includes the mean (*n =* 25) and CV (%).

Ecotype	Measures	FL (mm)	FWD (mm)	FWG (g)	PL (mm)	CD (mm)	SN	SL (mm)	SWD (mm)	SWG (g)	TSWG (g)	LL (mm)	LW (mm)	FS	CS
**Ecotype 1**	Mean	11.92	9.02	0.4377	17.29	4.77	8.28	4.00	3.04	0.0164	0.1241	36.63	17.25	1	2
	CV	10.03	6.82	15.43	17.06	5.78	40.29	10.65	12.99	27.75	22.49	10.24	13.17		
**Ecotype 2**	Mean	13.40	9.88	0.6835	19.58	4.68	7.32	4.33	3.28	0.0213	0.1384	28.93	12.70	2	2
	CV	12.39	9.32	22.87	17.93	8.99	36.51	8.96	16.13	17.77	38.86	10.27	33.48		
**Ecotype 3**	Mean	9.40	7.16	0.2822	20.78	4.86	4.28	4.07	3.08	0.0196	0.0770	38.17	14.90	1	1
	CV	8.29	8.61	21.39	17.88	4.83	41.31	7.02	11.34	14.75	34.31	12.02	10.93		
**Ecotype 4**	Mean	10.95	10.68	0.6500	21.12	4.60	6.6	4.02	2.92	0.0204	0.1179	45.19	18.45	2	3
	CV	16.00	8.44	25.14	20.22	10.63	61.08	8.36	14.51	17.39	49.02	13.47	9.68		
**Ecotype 5**	Mean	12.31	8.54	0.4515	19.50	5.12	3.32	3.74	2.99	0.0187	0.0628	46.21	18.00	3	2
	CV	5.87	8.61	18.71	19.29	10.98	44.10	8.17	12.01	17.71	38.46	9.13	11.28		
**Ecotype** 6	Mean	14.49	8.91	0.5162	18.29	3.87	6.96	4.67	3.02	0.0240	0.1486	38.30	13.17	2	1
	CV	7.57	5.46	12.80	15.70	15.51	31.99	8.45	13.25	22.43	22.08	13.49	13.50		
**Ecotype 7**	Mean	8.80	6.96	0.1998	21.29	4.46	2.96	3.36	2.78	0.0167	0.0390	36.45	14.44	2	1
	CV	12.08	13.43	36.19	11.76	10.28	37.11	8.44	13.26	103.16	37.34	13.70	29.27		
**Ecotype 8**	Mean	11.79	11.29	0.5942	16.06	3.73	10.08	3.40	2.50	0.0106	0.1022	33.36	12.27	3	3
	CV	5.39	17.54	15.31	24.48	18.80	29.75	20.43	13.08	17.18	25.17	11.26	15.47		
**Ecotype 9**	Mean	12.6	6.51	0.2334	20.6	4.24	2.28	3.95	3.13	0.0184	0.04	32.65	13.94	3	2
	CV	14.12	15.89	34.01	18.23	9.84	51.46	14.50	14.58	19.12	45.92	7.34	7.48		
**Ecotype 10**	Mean	16.22	7.52	0.4396	17.54	4.36	3.28	4.10	3.33	0.0201	0.0618	30.92	11.82	3	1
	CV	6.67	14.65	18.29	17.23	8.53	39.87	11.99	12.85	15.00	38.56	20.40	18.15		
**Ecotype 11**	Mean	13.86	7.19	0.3189	15.54	4.49	4.00	3.96	3.67	0.0206	0.0733	33.38	11.92	1	2
	CV	5.38	10.21	17.61	16.11	7.81	40.16	10.12	11.32	14.21	30.21	18.76	16.52		
**Ecotype 12**	Mean	16.24	8.27	0.4798	20.31	4.42	3.00	3.18	4.3	0.0212	0.0563	28.25	10.55	1	1
	CV	6.10	6.10	12.31	12.31	6.78	38.76	11.12	13.56	17.21	29.87	16.54	14.32		
**Ecotype 13**	Mean	12.98	6.18	0.2562	17.58	4.3	2.00	4.75	4.47	0.021	0.0381	30.44	13.6	1	2
	CV	5.41	1.23	10.29	11.23	7.56	40.97	8.76	13.21	16.79	29.01	16.70	15.20		
**Ecotype 14**	Mean	15.03	6.74	0.4252	19.12	4.97	4.00	4.47	3.9	0.0204	0.0704	31.86	11.58	1	1
	CV	6.35	2.43	15.16	14.56	6.78	38.72	8.11	14.21	16.72	19.67	14.32	13.29		
**Mean CV**		8.69	9.20	19.68	16.71	9.51	40.86	10.36	13.31	24.9	32.93	13.40	15.84		

Fruit length (FL), fruit width (FWD), fruit weight (FWG), peduncle length (PL), calyx diameter (CD), seed number (SN), seed length (SL), seed width (SWD), seed weight (SWG), total seed weight (TSWG), leaf length (LL), leaf width (LW), FS (fruits shapes): 1, elliptic; 2, obovate; 3, spherical, and CS (Calyx shapes): 1, closed; 2, partially opened; 3, opened.

**Table 2:** Correlation coefficients between the morphological traits studied.

	FL	FWD	FWG	PL	CD	SN	SL	SWD	SWG	TSWG	LL	LW
**FL**	1.000											
**FWD**	0.065	1.000										
**FWG**	0.442	0.874 *	1.000									
**PL**	−0.116	−0.368	−0.183	1.000								
**CD**	−0.294	−0.233	−0.167	0.311	1.000							
**SN**	0.129	0.845 *	0.738 *	−0.339	−0.368	1.000						
**SL**	0.562	0.0019	0.376	0.058	−0.019	0.115	1.000					
**SWD**	0.558	−0.432	0.003	0.256	0.388	−0.371	0.689 *	1.000				
**SWG**	0.395	−0.253	0.156	0.390	0.218	−0.285	0.849 *	0.730 *	1.000			
**TSWG**	0.320	0.695 *	0.814 *	−0.149	−0.183	0.819 *	0.616	0.060	0.305	1.000		
**LL**	−0.316	0.138	0.048	0.288	0.415	−0.090	−0.088	−0.272	0.143	0.017	1.000	
**LW**	−0.263	0.093	0.051	0.476	0.612	−0.0005	−0.085	−0.048	0.088	0.078	0.821 *	1.000

* Significant at *p*-value < 0.05.

**Table 3:** Correlation coefficients between the morphological and chemical traits studied.

	TA	TPC
**TA**	1	
**TPC**	0.632 *	1
**FWD**	0.679 *	0.509
**FL**	−0.541 *	−0.307
**FWG**	0.526	0.441
**PL**	0.272	0.043
**CD**	0.150	0.078
**SN**	0.598 *	0.562 *
**SL**	−0.109	−0.039
**SWD**	−0.467	−0.493
**SWG**	−0.217	−0.225
**TSWG**	0.562 *	0.545 *
**LL**	0.428	0.051
**LW**	0.458	0.159

* Significant at *p*-value < 0.05.

**Table 4:** Loadings of the studied morphological variables obtained in the PCA.

	PC1	PC2	PC3	PC4
**FWD**	0.332545	0.261105	0.016976	−0.250766
**FL**	−0.238809	0.374353	0.0722094	−0.23456
**FWG**	0.21349	0.378674	0.218042	−0.354937
**PL**	0.0300118	−0.332878	0.216556	−0.524048
**CD**	−0.041398	−0.268718	0.38474	−0.239024
**SN**	0.313662	0.312652	−0.0277603	0.0991843
**SL**	−0.113674	0.159997	0.440774	0.583266
**SWD**	−0.344552	0.0811821	0.103164	−0.126136
**SWG**	−0.204153	0.0776921	0.515538	−0.0716526
**TSWG**	0.249142	0.340977	0.292583	0.074471
**LL**	0.252782	−0.265527	0.291788	0.1241
**LW**	0.234908	−0.305274	0.306359	0.158535

**Table 5:** Loadings of the studied chemical variables obtained in the principal component analysis (PCA).

	PC1	PC2	PC3
**TPC**	0.276762	−0.215631	0.345485
**Delphinidin 3,5-*O*-diglucoside**	0.357497	−0.158785	−0.0858731
**Delphinidin 3-*O*-glucoside**	0.400637	0.0174325	0.0435637
**Cyanidin 3-*O*-galactoside**	0.230934	0.313119	−0.289586
**Cyanidin 3-*O*-glucoside**	0.340067	−0.0419079	0.366281
**Cyanidin 3-*O*-arabinoside**	0.026038	0.359961	0.483078
**Petunidin 3-*O*-glucoside**	0.330595	−0.247933	−0.143641
**Delphinidin 3-*O*-arabinoside**	0.102898	0.475758	0.0623436
**Peonidin 3-*O*-glucoside**	0.336837	0.0944947	0.286337
**Malvidin 3-*O*-glucoside**	0.157008	−0.280399	−0.360425
**Petunidin 3-*O*-arabinoside**	0.225104	0.385053	−0.246464
**Malvidin 3-*O*-arabinoside**	0.106503	0.403267	−0.30904
**TA**	0.38135	−0.111159	−0.165277

**Table 6:** Climatic and meteorological conditions in Puerto Real and San José del Valle.

Place	Altitude (m Above Sea Level)	T_max_ (°C) ^1^	T_min_ (°C) ^2^	Mean Annual Precipitation (mm)
Puerto Real	8	30	9	609
San José del Valle	141	34	6	720

^1^ T_max_ is the maximum average temperature of the warmest month. ^2^ T_min_ is the minimum average temperature of the coldest month.

**Table 7:** Morphological traits measured in *Myrtus communis* L. populations from Cadiz.

Morphological Traits	Code	Unit of Measure	Method of Measurement
Fruit traits			
Fruit length	FL	mm	Caliper
Fruit width	FWD	mm	Caliper
Fruit weight	FWG	g	Precision balance
Peduncle length	PL	mm	Caliper
Fruit shapes	FS	-	By observation: spherical, elliptic, obovate, pyriform, turbinate
Calyx shapes	CS	-	By observation: closed, partially closed, opened
Calyx diameter	CD	mm	Caliper
Seed traits			
Seed number	SN	-	Counter
Seed length	SL	mm	Caliper
Seed width	SWG	mm	Caliper
Seed weight	SWD	g	Precision balance
Total seed weight	TSWG	g	Precision balance
Leaves traits			
Leaf length	LL	mm	Caliper
Leaf width	LW	mm	Caliper

## Supplementary Materials

The following are available online at https://www.mdpi.com/2223-7747/8/9/328/s1, Table S1: Geographic characteristics of myrtle sampling ecotypes. For each ecotype, identity code and sampling location with geographical coordinates are reported.

## Abbreviations

BBD	Box–Behnken design
CD	calyx diameter
CS	calyx shapes
CV	coefficient of variation
FL	fruit length
FS	fruit shapes
FWD	fruit width
FWG	fruit weight
HCA	hierarchical cluster analyses
HPLC	high-performance liquid chromatography
LL	leaf length
LW	leaf width
MAE	microwave-assisted extraction
PCA	principal component analysis
PL	peduncle length
Q-ToF-MS	quadrupole-time-of-flight mass spectrometry
RSM	response surface methodology
SL	seed length
SN	seed number
SWD	seed width
SWG	seed weight
TA	total anthocyanins
TPC	total phenolic compounds
TSWG	total seed weight
UHPLC	ultra-high performance liquid chromatography
