# Supplementary material for: Discrimination of Myrtle Ecotypes from Different Geographic Areas According to Their Morphological Characteristics and Anthocyanins Composition
Source: Plants (Basel). 2019 Sep 5;8(9):328. doi: 10.3390/plants8090328 (PMC6784115; doi:10.3390/plants8090328)
Supplement: Supplementary file 1 [file plants-08-00328-s001.pdf]

Table S1. Geographic characteristics of myrtle sampling ecotypes. For each ecotype, identity code and sampling location with geographical coordinates are reported.

Code	Municipality	Latitude	Longitude	Collection Date
UCA-MY-1	Puerto Real	36.555310	−6.135020	30/11/16
UCA-MY-2	Puerto Real	36.555331	−6.135169	30/11/16
UCA-MY-3	Puerto Real	36.557085	−6.137332	30/11/16
UCA-MY-4	Puerto Real	36.557057	−6.137499	30/11/16
UCA-MY-5	Puerto Real	36.557134	−6.137815	30/11/16
UCA-MY-6	Puerto Real	36.557357	−6.137920	30/11/16
UCA-MY-7	Puerto Real	36.559021	−6.138241	30/11/16
UCA-MY-8	Puerto Real	36.557080	−6.134861	30/11/16
UCA-MY-9	San José del Valle	36.600369	−5.791030	29/11/16
UCA-MY-10	San José del Valle	36.603748	−5.793548	29/11/16
UCA-MY-11	San José del Valle	36.599750	−5.802470	29/11/16
UCA-MY-12	San José del Valle	36.603470	−5.788910	29/11/16
UCA-MY-13	San José del Valle	36.600850	−5.797750	29/11/16
UCA-MY-14	San José del Valle	36.599610	−5.794830	29/11/16
